# The Milk-Supply of Schools

**Published:** 1906-03-10

**Authors:** 


### The Hospital.

A Journal of

The Medical Sciences and Hospital Administration.

Vol. XXXIX.—No. 1,015. SATURDAY, MARCH 10, 1906.

### The Milk-supply of Schools.

The Consulting Medical Officer of Haileybury College, Dr. C. E. Shelley, has been engaged in a "preliminary inquiry" concerning the milk-supply of schools, an inquiry undertaken at the instance of the Council of the Medical Officers of Schools Association; and the results at which he has arrived have been issued in pamphlet form by the Association. In a paper read before that body by Dr. Christopher Childs, the Association was urged to formulate certain recommendations which, it was suggested, should be pressed upon the authorities of all schools; and the most important of these recommendations, because the one most open to discussion, was the institution of some method of sterilising milk delivered on the school premises before it was distributed for use. It was felt by the Council that this recommendation would certainly meet with a considerable amount of at least passive resistance, and that those who were responsible for it should place themselves in a position to meet all plausible objections. A headmaster was imagined who might be fully impressed with the importance of the question at issue, but who might ask to be informed of the actual facts bearing upon the position, and of the amount of school illness which has been traced to infection conveyed by milk. "I have known," such an one might say, "the life of my school for such and such a number of years: we have had from time to time epidemics of such and such diseases; but they have all (or almost all) been ascribed to personal infection, the details of which appeared at the time to be clearly established. None of this illness has been traced to milk. Are we a fortunate exception to a general rule; and, if so, to what extent are we exceptional? Further, what are the results achieved in those schools in which milk is sterilised, as compared with those in which it is drunk *au naturel*?"

To such questions as these the Council of the Association was not prepared with any detailed or convincing answers; and, in the hope of obtaining them, forms of inquiry bearing upon the subject were issued to every member of the Association, with an assurance that the answers would be employed for statistical purposes only, and not in any such way as might render it possible to identify them with any particular school. More than one hundred circulars of inquiry were sent out, and replies were received to forty-three, including one from the University of Oxford.

Of the forty-three replies, forty-one afforded matter for tabulation, and the results are briefly stated by saying that they cover 203,385 yearly observations on some 33,894 individual pupils whose ages ranged from four to nineteen and a half years. Among these there are reported to have occurred, as effects of milk supplied to the schools, twenty cases of enteric fever, ten of diphtheria, fifty of diarrhœa, and seventy-five of septic sore throat, the gross total of assumed milk disease being, therefore, less than 0.045 per cent. of the pupils, and the total of the two first-named and more serious diseases being only 0.0088 per cent. It would be very hazardous to assume that the milk was *proved* to be the source of infection in all these cases; and it is manifest that the figures afford too slender a basis for the erection of any substantial superstructure. It is rendered still more slender by the information that the group of twenty cases of enteric fever were traced to the eating of strawberries and *cream* at a shop belonging to the dairy-farm which supplied the school, and which was shown at the time to be the source of several other cases in the town, while the milk supplied from the same dairy to the school does not seem to have given rise to illness. There is at least one well-known instance of an epidemic produced by the cream consumed at a large evening entertainment, and Dr. Shelley points out that a relatively excessive virulence of the *cream* obtained from milk infected with the *Bacillus typhosus* seems to be strongly suggested by accumulating observations. In the case of the school, the report furnishes no evidence that the cream was derived from the same source as the milk supplied to the scholars; and it is at least conceivable that the purveyor may have taken more care with regard to milk delivered to a regular and important customer than with regard to cream sold to casual purchasers at his shop. However this may be, it seems to us that the report justifies the opinion that, with such reasonable precautions as every schoolmaster would be practically certain to take, infection from milk may almost be regarded as a negligible quantity, so far, at least, as enteric fever, scarlet fever, and diphtheria are concerned. By "reasonable precautions" in this sense we mean securities for the health of the cows, the cleanliness of the dairy and the attendants, the freedom from contamination of the water, and the protection of the milk against infection in transit. With regard to the communication of tuberculosis we are on less assured ground, For better knowledge on this part of the subject it will be necessary to wait for the report of the Royal Commission now sitting and collecting evidence. In the event of this Commission declaring the sterilisation of milk by heat to be a measure of necessity, it must still be remembered that the bacillus of tubercle may be killed by a temperature much below the boiling-point of milk, say, 150° or 155° F., and that this temperature has very little influence upon flavour. How far it may affect nutritive value has yet, perhaps, to be ascertained.

